# From Grape Stalks to Lignin Nanoparticles: A Study on Extraction Scale-Up, Solubility Enhancement and Green Nanoparticle Production

**DOI:** 10.3390/foods14244274

**Published:** 2025-12-12

**Authors:** Ana C. Cassoni, Ana I. Bourbon, Lorenzo Pastrana, Marta Vasconcelos, Manuela Pintado

**Affiliations:** 1 CBQF—Centro de Biotecnologia e Química Fina—Laboratório Associado, Escola Superior de Biotecnologia, Universidade Católica Portuguesa, Rua Diogo Botelho 1327, 4169-005 Porto, Portugal; acassoni@ucp.pt (A.C.C.); mvasconcelos@ucp.pt (M.V.); 2 International Iberian Nanotechnology Laboratory (INL), Av. Mestre José Veiga s/n, 4715-330 Braga, Portugal; ana.bourbon@inl.int (A.I.B.); lorenzo.pastrana@inl.int (L.P.)

**Keywords:** lignin, agrofood, byproducts, solubilization, nanoparticles

## Abstract

To effectively valorize lignin, some challenges must be addressed. First, emerging techniques based on green methods may experience difficulties during the scale-up process. Secondly, the low solubility of lignin can hinder further valorization. This study investigated the impact of lignin extraction scale-up on yield and purity and evaluated strategies to enhance lignin solubility. Lignin from grape stalks was extracted using two previously optimized methods—alkaline and deep eutectic solvents—at a scale-up by factors of 5, 10, and 20 times. Although a slight decrease in extraction yield was observed with increasing scale, lignin purity remained consistent across all conditions. After extraction, lignin samples were subjected to solubilization tests using surfactants (Tween 20, Tween 80, and polyethylene glycol) and organic solvents (ethanol and acetic acid). Results demonstrated that surfactants were notably more effective in solubilizing lignin (up to 74.5%) compared to organic solvents. Furthermore, as an alternative to lignin solubilization, the production of lignin nanoparticles through ultrasonication with minimal chemical use was also explored. Upon optimization, spherical nanoparticles with a mean diameter of approximately 200 nm were successfully obtained. The use of surfactants was necessary to avoid nanoparticle aggregation during concentration steps and to enhance colloidal stability. This study demonstrates the feasibility of scaling up lignin extraction methods and further explores two approaches to enhancing the valorization of the obtained lignin—solubilization and the production of lignin nanoparticles—thereby contributing to the development of efficient and sustainable strategies for diverse lignin-based applications.

## 1. Introduction

Lignin is a complex aromatic biopolymer present in lignocellulosic biomass, along with cellulose and hemicellulose [1,2]. It is the second most abundant natural polymer, after cellulose, and therefore represents a significant opportunity for valorization [2]. Agro-food residues can be valuable sources of lignin, and their extraction contributes to the circular economy by reducing waste and creating new high-value products. In particular, the present study uses grape stalks, an abundant residue, and previous studies have reported good yields and purity in lignin extraction [3].

To effectively valorize lignin, several challenges need to be addressed. First, there is a need for effective extraction methods that consider the variability of lignin sources [4,5]. Additionally, the efficiency of scaling up extraction methods is crucial for industrial implementation and economic viability [6]. While some industrial methods have already been implemented, such as kraft, soda, sulfite, and organosolv [7], various other extraction methods are also available at lab and biorefinery scales. These include emerging green methods (e.g., ionic liquids, deep eutectic solvents (DES)), and more research on their scalability is needed [6,8]. Process optimization, technological innovations, and life cycle analysis are some of the steps that are essential in the scale-up process [9].

Limited lignin solubility is also a major challenge that significantly affects its potential applications. Due to its complex structure and hydrophobic nature, lignin has limited solubility in most solvents and, in general, harsh chemicals are required, which may impair its utilization [10]. Surfactants have been explored as solubilizing agents with promising results [11,12], but the solubilization behavior of different lignins remains poorly understood.

As an alternative to solubilization, lignin nanoparticles have emerged as a promising approach due to their unique properties and diverse applications across fields, such as biomedicine, composites, and controlled delivery, among others [7,13,14]. Several methods for producing nanoparticles have been developed, including solvent exchange, antisolvent precipitation, and emulsion [15,16]. Sonication, a simple, straightforward, and cost-effective method, is being explored with promising results [17,18,19].

The present study aims to address these challenges by investigating (1) the scale-up of lignin extraction from grape stalks, using both alkaline and deep eutectic solvent methods, (2) the solubilization of the extracted lignins in various solvents, and (3) the production of lignin nanoparticles through sonication.

## 2. Materials and Methods

Grape stalks of the Vinhão variety were kindly provided by Quinta de Mascate (Vila Verde, Portugal). Fresh biomass was immediately stored at −20 °C. During experiments, biomass was then dried, milled, and stored at room temperature. High purity reagents used in this work were purchased from Merck KGaA (Darmstadt, Germany)—sodium hydroxide (NaOH), sulphuric acid (H_2_SO_4_), lactic acid (LA), Tween 20 (T20), Tween 80 (T80), and polyethylene glycol (PEG); VWR (Radnor, PA, USA)—dimethyl sulfoxide (DMSO), ethanol, methanol, acetic acid; and TCI Chemicals (Tokyo, Japan)—choline chloride (CC).

### 2.1. Scale-Up

Scale-up tests were performed at 5, 10, and 20 times the original scale (1 g of residue in 10 mL of solvent) using previously optimized alkaline and deep eutectic solvent extraction methods [3] . Briefly, the alkaline extraction method was carried out with 6% NaOH solution at 80 °C for 1 h, while the DES extraction utilized a mixture of lactic acid and choline chloride (5:1 molar ratio) at 120 °C for 5 h. There were two modifications to the filtration steps. In the first filtration step, which separates the biomass from the dissolved lignin, a cloth filter was employed instead of centrifugation. For the second filtration step, which recovers the lignin precipitate, vacuum filtration was used with 24 mm Whatman grade 113 qualitative filter paper (Cytiva, Marlborough, MA, USA) in place of centrifugation. After the second filtration, the lignin precipitates were thoroughly washed with deionized water until the wash water reached neutral pH, ensuring the removal of any remaining chemicals. Lignin was dried at 40 °C until constant weight. Lignin purity was determined following National Renewable Energy Laboratory (NREL) protocol [20]. Lignin yield was estimated as follows:

$$Lignin\;Yield\left(\%\right)=\frac{\left(mass\;of\;precipitated\;lignin\right)}{\left(mass\;of\;lignin\;in\;biomass\right)}\times 100$$

### 2.2. Lignin Solubilization

Before the solubilization tests, lignin samples were micronized using a Planetary Ball Mill PM 100 (Retsch GmbH, Haan, Germany). Alkaline lignin (AkL) was processed for 5 min and deep eutectic solvents lignin (DESL) for 20 min, until a homogenous fine powder was obtained. Micronization times were optimized for each sample to achieve similar levels of homogeneity, rather than standardizing the processing time across both samples. Tests with non-micronized lignin were also performed for comparison purposes.

A calibration curve was prepared using lignin samples dissolved in 10% (*v*/*v*) dimethyl sulfoxide (DMSO) at a concentration of 2 mg/mL, under constant agitation at 100 rpm for 24 h to ensure maximum dissolution. After agitation, the samples were centrifuged to separate undissolved lignin. The supernatant was then diluted to a series of 10 concentrations (±0.09–0.9 mg/mL), and absorbance was measured spectrophotometrically using a Shimadzu UV-Vis spectrophotometer (mini-1240, Kyoto, Japan) at 280 nm [21], with absorbance values ranging from 0.1 to 0.999. The undissolved lignin from the centrifugation step was dried and weighed to determine the insoluble fraction. The calibration curve was established by plotting absorbance values against the corresponding lignin concentrations (Supplementary Material—Figure S1).

Solubility tests were evaluated with a total of five solvents: three surfactants (T20, T80, and PEG) and two organic solvents (ethanol and acetic acid), all prepared at a concentration of 10% (*v*/*v*). Lignin samples were added to each solvent at a concentration of 2 mg/mL and subjected to constant agitation at 100 rpm for 24 h to ensure maximum solubilization. Subsequently, samples were centrifuged to separate undissolved lignin, and the supernatant was analyzed spectrophotometrically using a Shimadzu UV-Vis spectrophotometer (mini-1240, Kyoto, Japan) at a wavelength of 280 nm [21]. Solubilized lignin concentration was determined by interpolating the measured absorbance values using the previously established calibration curve.

### 2.3. Lignin Nanoparticles

#### 2.3.1. Lignin Nanoparticle Production

Lignin nanoparticles were produced by ultrasonication using a “never-dried lignin approach”, as described by Agustin et al. [17]. Freshly extracted lignin was used directly after extraction, without any drying steps. To determine the lignin concentration, the dry weight of the never-dried lignin was determined.

The ultrasonication optimization was carried out using an Ultrasonic Processor Sonics VCX 130 (Sonics & Materials Inc., Newton, CT, USA) with a 6 mm tip. Lignin dispersions were prepared by dissolving different concentrations of lignin (0.25, 0.5, and 1% *w*/*v*) in 10 mL of deionized water and agitating overnight to ensure sample homogeneity. Then, ultrasonication was tested at 80% amplitude for 15, 30, and 60 min. To prevent sample overheating and potential degradation of the lignin structure, samples were placed in an ice bath, and ultrasonication was performed in pulses (10 s on, 10 s off).

Once the parameters were optimized, scale-up tests (5-fold) were conducted using the Ultrasonic processor Q700X (Qsonica, Newtown, CT, USA) with a 10 mm tip. The samples were maintained in an ice bath, under constant agitation, and the temperature was monitored to ensure it did not exceed 45 °C.

#### 2.3.2. Lignin Nanoparticle Concentration and Stability

After nanoparticle production, the samples were placed in an oven at 45 °C to evaporate the water and concentrate the nanoparticle solution (2 and 5-fold). At this stage, two surfactants (T20 and T80) were tested at 0.05% during nanoparticle production to investigate their impact on nanoparticle size, morphology, and aggregation with increasing concentration.

Lignin nanoparticles were stored at 4 °C, and their stability was evaluated biweekly using dynamic light scattering (DLS) with a Zetasizer Nano ZSP (Malvern Panalytical Ltd., Malvern, UK) and monthly using transmission electron microscopy (TEM) over a 60-day period.

#### 2.3.3. Lignin Nanoparticle Characterization

Lignin nanoparticle samples were analyzed directly after sonication without prior processing using DLS with a Zetasizer Nano ZSP (Malvern Panalytical Ltd., Malvern, UK), which assessed particle size, polydispersity index (PDI), and zeta potential (ZP). The instrument employed Non-Invasive Back-Scatter (NIBS) technology, which detects scattered light at a fixed angle of 173°. Measurements were performed using a disposable folded capillary cell (Malvern Panalytical Ltd., Malvern, UK) at a constant temperature of 25 °C. Particle size values presented in this study are based on the Z-average, which is calculated from the intensity of light scattered by each particle fraction or family, expressed as a percentage. Data were acquired and analyzed using Zetasizer v. 7.11 software. All measures were performed in duplicate with independently prepared samples.

The morphology of the lignin nanoparticles was evaluated by transmission electron microscopy (TEM) (JEOL JEM 2100—HT—80–200 kV LaB6 gun, JEOL Ltd., Tokyo, Japan). The images were digitally recorded using an UltraScan^®^ 4000 CCD camera (Oneview, Gatan, Pleasanton, CA, USA). The aliquots with a 10 μL suspension of emulsion were deposited onto grids coated with an ultrathin carbon film (400 mesh, approx. grid hole size of 42 μm, PELCO^®^, TED PELLA Inc., Redding, CA, USA), and UranyLess EM Stain (Electron Microscopy Sciences (EMS), Hatfield, PA, USA) was used as the contrast agent.

Lignin nanoparticle samples were centrifuged and dried to be analyzed by Fourier Transformed InfraRed (FTIR) in an ABB MB3000 FT-IR spectrometer (ABB, Zürich, Switzerland) equipped with a horizontal attenuated total reflectance (ATR) sampling accessory (PIKE Technologies, Madison, WI, USA) with a diamond/ZnSe crystal. Each lignin sample was subjected to eight scans, and spectrums were recorded from 4000 to 600 cm^−1^.

### 2.4. Statistical Analysis

Statistical analysis was performed by one-way ANOVA, carried out in IBM SPSS Statistics (v26). Results are reported as means ± standard deviation (S.D.), and significant differences were considered at *p* < 0.05.

## 3. Results and Discussion

### 3.1. Scale-Up

Table 1 summarizes the yield and purity results obtained from the lignin extraction scale-up tests using the alkaline and deep eutectic solvent methods.

The scale-up tests for the alkaline and DES methods yielded different results. For the alkaline method, no significant differences in yield and purity were observed among the various scale-up factors (5-, 10-, and 20-fold). Moreover, when compared to the original method optimized by Cassoni et al. [3], both yield and purity were maintained (Table 1). For the DES method, a progressive decrease in lignin yield was observed with increasing scale-up fold, and significant differences were observed compared with the original method (Table 1). This decrease in yield can be attributed to modifications made to the second filtration step during scale-up. During vacuum filtration, the DESL exhibited strong adhesion to the filter paper, hindering full recovery of the lignin. In the alkaline method, lignin was less adherent, allowing for better recovery.

It is common to observe differences in lignin yield when scaling up extraction processes, as the methods and equipment used often need to be adapted to more biomass and subsequently larger volumes [22]. However, given that the scale-up factors in this study were relatively small and still within the laboratory scale, the differences in yield should not be substantial. The observed decrease in yield for the DES method can primarily be attributed to challenges encountered during the filtration step, rather than to the increase in biomass quantity or the method’s inherent scalability.

Despite the differences in yield, the purity of the extracted lignin remained consistent across all scale-up factors for both the alkaline and DES methods, with no significant differences observed. This suggests that the modifications made to the method during scale-up did not compromise the quality of the extracted lignin.

Recent studies have yielded promising results in the scale-up processes of lignin extraction from agricultural and food sources. Chen et al. [23] performed a 100-fold scale-up of alkaline extraction (Soda process) from rice straw, achieving a slightly lower yield than the lab-scale process. Novakovic et al. [24] successfully tested alkaline delignification of wheat straw on a 100-fold scale-up, maintaining extraction efficiency. Cequier et al. [25] performed a 67-fold scale-up of lignin extraction from olive pomace using ionic liquids and were able to retain a yield of lignin (~40%). These studies highlight the potential for the successful scale-up of lignin extraction methods from diverse agro-food sources. The ability to maintain yield and purity at larger scales is crucial for the industrial application of these processes. Variations in efficiency can be attributed to differences in method parameters and equipment.

### 3.2. Lignin Solubilization

**Figure 1:**
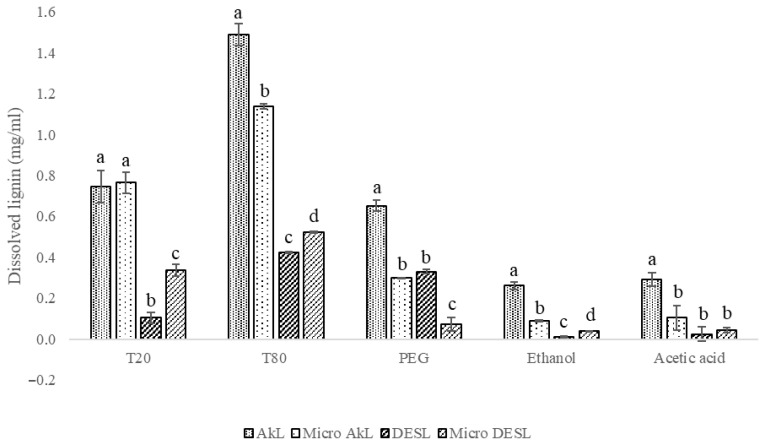
Solubilization/dissolution of lignin and micronized lignin samples (AkL and DES) using surfactants (T20, T80, PEG) and organic solvents (ethanol and acetic acid). Different superscript letters represent a significant statistical difference (*p* < 0.05) within the solvent.

The solubility of four lignin samples, namely alkaline lignin (AkL), DES lignin (DESL), and their micronized versions (micro-AkL and micro-DESL), was investigated using surfactants and organic solvents at 10% (Figure 1). The low solvent concentration aligns with the study’s objective of developing a greener, more sustainable approach, although the authors acknowledge the solubility limitations associated with such low concentrations. The micronization process was employed to increase the surface area of the lignin samples and evaluate its effect on dissolution. The selection of surfactants was based on the hydrophobic nature of lignin, while organic solvents were chosen due to their compatibility and low toxicity [10].

The results suggest that surfactants exhibit greater overall solubility than organic solvents, as expected given the structure and properties of lignin. Among the three surfactants tested, T80 demonstrated the highest effectiveness in dissolving all four lignin samples, with the most significant solubility observed for AkL (1.49 ± 0.06 mg/mL) and micro-AkL (1.14 ± 0.01 mg/mL). T20 also showed reasonable dissolution of AkL samples, with solubility of approximately 0.75 mg/mL. Previous research showed results aligned with our findings: Melro et al. [12] studied the effect of surfactants on kraft lignin dissolution and found that T20 (0.5 mol/L) allowed for 100% dissolution, while Xu et al. [11] reached >70% using T80 at a mass ratio of 0.76:1 (H_2_O:T80). Although these studies reported higher dissolution rates, they employed significantly higher surfactant concentrations (approximately 60%). Our approach achieved a maximum dissolution of 74.8% for AkL and 26.2% for DESL, while using substantially less surfactant (10%), suggesting improved dissolution efficiency.

Comparing the lignin samples, AkL exhibited better solubility across all solvents compared to DESL. Surprisingly, the micronized AkL had significantly lower solubility than non-micronized AkL, except with T20, which showed similar solubility for both samples. The reduced solubility of micronized AkL suggests that the micronization process may have induced changes in the lignin structure that hindered its dissolution. Specifically, micronization may have led to particle agglomeration due to stronger inter-particle forces [26] or possible surface chemistry changes [27,28]. However, further experimental validation is needed to confirm and elucidate the underlying mechanisms. In contrast, the micronization process enhanced the solubility of DESL in almost all solvents, except for polyethylene glycol (PEG). This enhanced solubility might be due to the increased surface area and improved dispersion [28] of micronized lignin. The contrasting behavior between AkL and DESL reinforces that the extraction method significantly influences lignin’s properties and how micronization affects dissolution. These findings show the importance of considering the interaction between extraction methods and post-processing techniques, such as micronization, when developing strategies for lignin valorization.

### 3.3. Lignin Nanoparticles

#### 3.3.1. Lignin Nanoparticle Production

The dry weight tests conducted to determine the concentration of lignin showed no significant differences between AkL and DESL, despite the clear visual and textural differences observed in the “never-dried lignin” (AkL: 18.7 ± 1.03%; DESL: 20.1 ± 1.43%). This information was crucial for controlling lignin concentration during nanoparticle formation and ensuring reproducibility across assays.

Several ultrasonication tests were conducted to determine the optimal conditions for the lignin nanoparticle production. Ultrasonication parameters, including amplitude, time, and lignin load, were tested to provide insights into their roles in controlling lignin nanoparticle size and uniformity. The main objective was to minimize particle size while considering time and energy consumption. Higher amplitudes were necessary to achieve smaller particles within shorter ultrasonication times. Lignin load also influenced nanoparticle size, with AkL at 1% lignin load resulting in particles of 283.3 ± 4.3 nm and DESL at 0.5% load yielding nanoparticles ranging from 400 to 500 nm, which is considerably large. Although longer ultrasonication times (60 min) yielded the smallest particle size (180.37 ± 3.01 nm), the reduction in particle size did not justify the increased time and energy consumption required for the 60 min ultrasonication process. Hence, the optimal conditions were established as follows: 80% amplitude, 15 min, and 0.5% load for AkL (214.27 ± 1.17 nm); and 80% amplitude, 15 min, and 0.25% load for DESL (185.1 ± 1.56 nm). These optimized parameters allowed us to achieve small particle sizes while maintaining process efficiency. PDI values were below 0.200 for all tests, indicating uniform particle size.

Upon optimization, scale-up tests were conducted (5-fold), and the particle sizes were similar to those obtained in the optimization: AkL—225.73 ± 3.15 nm; DESL—178.57 ± 5.53 nm. The PDI values of 0.148 ± 0.01 for AkL and 0.233 ± 0.03 for DESL indicate that the nanoparticles are uniform in size. Thus, the scale-up was successfully achieved without compromising nanoparticle formation.

Agustin et al. [17] developed a method for producing nanoparticles using “never-dried lignin” through sonication, combined with acid precipitation. The authors successfully produced nanoparticles with sizes less than 100 nm using a 5 min sonication time. Camargos et al. [29] also used never-dried lignin at a 0.1% load and tested sonication times of 15–90 min. The obtained nanoparticles ranged from 170 to 435 nm. Moreover, a surfactant was added to investigate its effect on the particles. The authors concluded that for sonication times shorter than 30 min, the surfactant favored nanoparticle production by reducing aggregation and particle size. The findings of the present study are consistent with those of previous studies.

In contrast, studies using dried lignin dispersions have reported longer sonication times to achieve similar nanoparticle sizes. Gonzalez et al. [30] performed ultrasonication of a 0.1% dried lignin dispersion for 2, 4, and 6 h. The authors observed that longer sonication times resulted in smaller particle sizes, ranging from 10 to 50 nm. Similarly, Gilca et al. [18] used a dried lignin dispersion with a sonication time of 60 min and produced nanoparticles with sizes ranging from 100 to 200 nm. Pérez-Rafael et al. [31] tested 2 h of sonication, with 20 min sampling intervals, and the results showed an exponential decrease in nanoparticle size over time, starting at 2500 nm and reaching sizes smaller than 700 nm at 2 h. However, results showed high PDI values. Edmundson et al. [19] performed a 3 h sonication on 0.5% lignin dispersions and obtained ~200 nm particles.

Comparing the results from studies using never-dried lignin and dried lignin dispersions, it is possible to notice that using never-dried lignin allows for the production of smaller nanoparticles in significantly less time. In contrast, dried lignin dispersions require longer sonication times (over 60 min), which is relevant in terms of time and energy consumption.

#### 3.3.2. Lignin Nanoparticle Concentration

Considering the defined lignin loads, the concentrations of lignin nanoparticles were determined to be 0.94 ± 0.05 mg/mL for AkL and 0.5 ± 0.04 mg/mL for DESL. To increase the concentration of lignin nanoparticles, evaporation was selected as the preferred method, as alternative approaches such as centrifugation or lyophilization have been previously shown to induce particle agglomeration and/or aggregation [32,33]. However, the concentration via evaporation also led to visible aggregation, mainly for DESL. Hence, the effect of surfactants (T20 and T80) on preventing particle aggregation during evaporation-based concentration [33] was investigated. Table 2 presents the particle sizes and PDI for the non-concentrated and concentrated samples with surfactants.

Both T20 and T80 show similar stabilizing effects, although T80 samples present slightly smaller particle sizes across most conditions (Table 2). AkL nanoparticles seem to be more stable upon concentration, maintaining consistent particle sizes regardless of the concentration factor. In contrast, DESL nanoparticles showed differing stability depending on the surfactant used. With T80, DESL particles maintained reasonable stability even at higher concentrations, whereas with T20, a substantial increase in both size (329.1 ± 1.10 nm) and PDI (0.308 ± 0.03) was observed at a 5-fold concentration, indicating destabilization.

Lignin nanoparticles showed PDI values below 0.2, confirming relatively monodisperse particle samples. The exception was the DESL/T20 combination at a 5-fold concentration, which exhibited higher polydispersity, suggesting aggregation or colloidal instability under these conditions.

These results demonstrate that particle aggregation can be effectively prevented through the addition of minimal quantities of surfactant (0.05%), primarily at a 2-fold concentration (Table 2). Although 5-fold concentrated samples exhibited acceptable average particle sizes according to DLS measurements, some peaks with larger sizes appeared in the scan, and there was observable particle aggregation. Hotze et al. [33] reported the role of surfactants in stabilizing nanoparticles during concentration processes. The current study extends these findings by specifically showing the effect of T20 and T80 on the stability of lignin nanoparticles.

**Figure 2:**
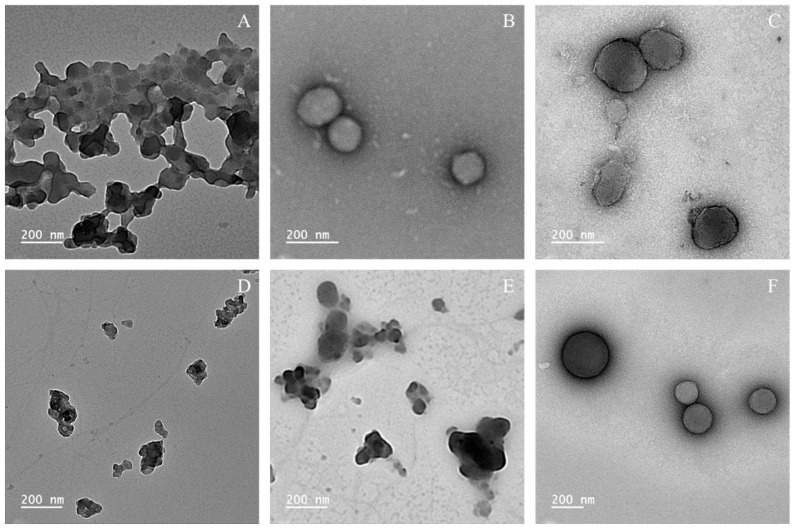
TEM images of lignin nanoparticles at 2-fold concentration. (**A**) AkL particles produced with H_2_O; (**B**) AkL particles produced with T20; (**C**) AkL particles produced with T80; (**D**) DESL particles produced with H_2_O; (**E**) DESL particles produced with T20; (**F**) DESL particles produced with T80.

In addition, particles produced with surfactants showed increased sizes compared to those prepared in water alone. To confirm particle size and morphological characteristics, TEM analysis was performed (Figure 2).

Considering the TEM images shown in Figure 2, it is observed that the overall particle sizes are smaller than those obtained by DLS measurements, as expected, with sizes of approximately 200 nm or less. This could be attributed to the limitations of DLS measurements, which detect the hydration layer surrounding the nanoparticles, potentially leading to an overestimation of particle dimensions [34]. Moreover, although polydispersity is low, multiple scattering effects may also have played a role in the overestimation of particle size by DLS, since the samples were measured directly after sonication [35]. Through the images, it is clear that particle aggregation occurred in the water samples and DESL with T20, corroborating the previously discussed results. AkL particles with T20 and T80 have similar sizes and morphology, with an irregular spherical shape and minimal aggregation. DESL with T80 stands out as the best sample, with spherical particles with little to no aggregation.

Studies that used “never-dried lignin” reported varying morphologies. Agustin et al. [17] obtained spherical nanoparticles by combining sonication with acid precipitation. As reported by Camargos et al. [29], nanoparticles were heterogeneous, presenting a flat, granular shape. They noted that increasing sonication time led to more homogeneous nanoparticles. Even with the addition of surfactant, Camargos et al. [29] still observed aggregates, and the morphology remained similar. The results are comparable to our study, as we also observed aggregates and heterogeneous shapes. However, when using surfactants, we achieved quasi-spherical and spherical nanoparticles with little to no aggregation. In addition, our method is more straightforward than that used by Agustin et al. [17], as we did not use acid precipitation and still obtained high-quality nanoparticles with minimal chemicals.

In contrast, studies using dried lignin dispersions have reported irregular-shaped nanoparticles [15,18,30,36]. These findings reiterate that using dried lignin dispersions is less effective than the method employed in the present study.

#### 3.3.3. Lignin Nanoparticle Stability

**Figure 3:**
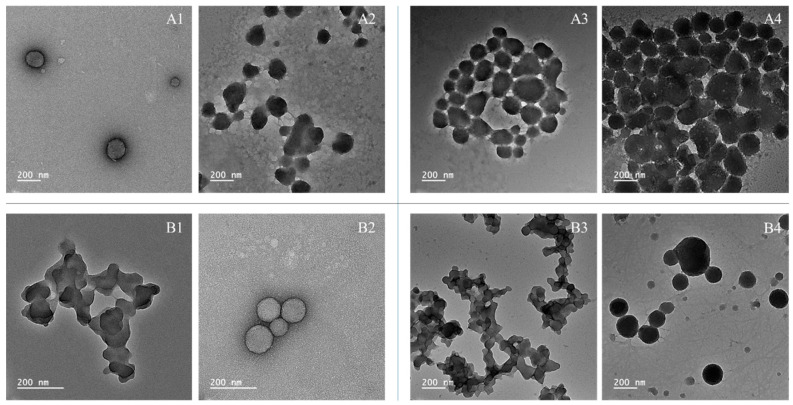
TEM images of lignin nanoparticles across 60 days of cold storage. (**A1**) 30-day storage of AkL/T20; (**A2**) 30-day storage of AkL/T80; (**A3**) 60-day storage of AkL/T20; (**A4**) 60-day storage of AkL/T80; (**B1**) 30-day storage of DESL/T20; (**B2**) 30-day storage of DESL/T80; (**B3**) 60-day storage of DESL/T20; (**B4**) 60-day storage of DESL/T80.

The lignin nanoparticle (2-fold concentration) stability results, according to DLS measurements, did not show significant differences in size and PDI throughout 60 days of cold storage, with particle sizes ranging from 200 to 300 nm and PDI values below 0.300 (Table 3). However, TEM images show loss of stability, with morphological changes and increased aggregation (Figure 3), mainly at 60 days of storage.

After 30 days of storage, AkL/T20 and DESL/T80 lignin nanoparticles maintained their morphology and showed reduced aggregation. At 60 days’ storage, DESL/T80 showed the best stability among the samples. Although loss of stability was evident, it is possible to observe that the particles maintained their spherical shape, and the aggregation was considerably lower compared to the other samples.

Colloidal stability was also investigated by measuring zeta potentials (Table 3). For AkL nanoparticles, the initial zeta potentials were −26 mv for T20 and −30.7 mV for T80. After 60 days of storage, the zeta potential decreased to −25.6 mV and −16.5 mV, respectively. For DESL nanoparticles, the initial zeta potentials were −27.1 mV for T20 and −33.2 mV for T80. At 60 days of storage, the zeta potential decreased to −22.6 mV and −27.6 mV, respectively. Although the decrease in zeta potential was not substantial for most samples (except for Ak with T80), the final values were below −30 mV. A zeta potential with an absolute value greater than 30 mV generally indicates good colloidal stability, as the aggregation is prevented by the electrostatic repulsion between particles [37,38]. A zeta potential of about 20 mV is considered to provide short-term stability [38]. Therefore, the observed zeta potential corroborates that the produced lignin nanoparticles lose colloidal stability over time, which leads to aggregation and sedimentation. DESL with T80 still shows the best stability, with −28 mV at 60 days of storage, indicating greater resistance to aggregation than the other samples.

Camargos et al. [29] reported zeta potentials more negative than −30 mV, indicating good colloidal stability. However, there was no data on long-term storage stability. Agustin et al. [17] reported that a high negative charge was crucial for achieving great stability for 180 days, mainly at 4 to 7 pH values. At a lower pH, there was a loss of stability and increased agglomeration. Edmundson et al. [19] also reported higher absolute zeta potentials for alkaline lignin nanoparticles (−48 to −60 mV), which corroborates that a higher pH contributes to the stability of nanoparticles. The pH of lignin nanoparticle solutions in our study was around 4.5 to 5, which may have influenced stability. Gonzalez et al. [30] were able to achieve stable lignin nanoparticles (6 h sonication) for 5 months without the formation of solid precipitate.

The present work showed that lignin nanoparticles’ stability is lower than that reported in the literature (maximum 60 days for DESL/T80). This could be due to several factors, since the stability of nanoparticles can be affected by the lignin source, extraction method, sonication process, and storage conditions [13,15,39]. Our study reveals that different extraction methods (alkaline vs. deep eutectic solvents) yield nanoparticles with distinct shapes and stabilities, regardless of the lignin source, nanoparticle production process, or storage conditions. This finding highlights the significant impact of the extraction method on the properties and stability of the resulting lignin nanoparticles. Additionally, investigating the effect of pH on the stability of the nanoparticles, as highlighted by Agustin et al. [17], could provide insights into enhancing long-term stability.

#### 3.3.4. FTIR

**Figure 4:**
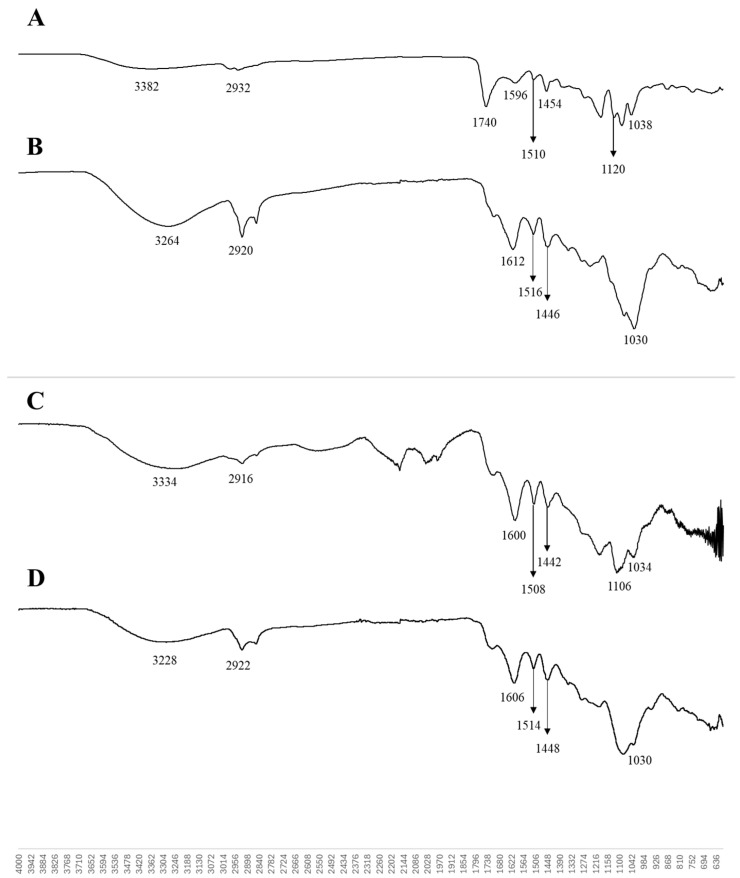
FTIR spectra of AkL (**A**), AkL nanoparticles produced with T80 (**B**), DESL (**C**), and DESL nanoparticles produced with T80 (**D**).

The FTIR spectra of the AkL and DESL nanoparticles (produced with T80) are very similar and show some changes in the structure of lignin after ultrasonication, when compared to the lignin structure before the ultrasonication process (Figure 4). Both spectra show typical wide bands (3264 cm^−1^; 3288 cm^−1^) associated with the O-H stretching of hydroxyl groups, followed by bands related to methyl group C-H stretching (2920 cm^−1^; 2922 cm^−1^). A loss of intensity of the C=O stretching band (1740 cm^−1^) is observed, mainly in the AkL sample. The band 1640 cm^−1^, which is reported to increase intensity with ultrasonication [17,30], was observed only in the AkL sample, indicating some degree of oxidation. Typical bands corresponding to the aromatic skeletal vibration are present (1612, 1606, 1516, 1514 cm^−1^) [18,40,41]. The C-H bending vibration of methyl and methylene groups is observed on 1446 and 1448 cm^−1^ bands [42,43]. Stronger and less defined peaks were observed in the 1010–1030 region, which could be due to C−O deformation in methoxyl groups [17]. Bands 1030 cm^−1^ are related to the guaiacyl ring [42].

## 4. Conclusions

The scale-up of lignin extraction from grape stalks using alkaline and deep eutectic solvent methods was investigated, along with the solubilization of the extracted lignins and the production of lignin nanoparticles. The alkaline extraction method was scaled up 20-fold without changes in yield or purity, whereas the DES method showed a decrease in yield due to filtration challenges, while purity was maintained. The filtration steps appeared to be crucial in the efficiency of the lignin extraction, and therefore, scale-up filtration optimization is required.

The solubility tests showed that surfactants, particularly T80, were more effective at dissolving lignin than were organic solvents. However, for the same solubility method, alkaline lignin exhibited higher solubility than DES lignin. Micronization improved the solubility of DES lignin but decreased the solubility of alkaline lignin, reinforcing that the extraction method affects lignin properties. The use of surfactants as a less-harmful alternative to organic solvents for lignin solubilization could expand the range of lignin applications while minimizing environmental impact. Lignin nanoparticles were successfully produced using “never-dried lignin” and ultrasonication, and the process was scaled up without changes in particle size and morphology. The addition of surfactants prevented particle aggregation during nanoparticle concentration, and TEM analysis confirmed that quasi-spherical and spherical morphologies with minimal aggregation were achieved. DESL nanoparticles produced with T80 exhibited the best stability (60 days), maintaining their spherical shape and showing lower aggregation than other samples. The production of lignin nanoparticles is another valorization route that uses minimal amounts of chemicals to maintain the process’s green nature.

These findings contribute to the development of sustainable, eco-friendly, and innovative lignin valorization approaches, promoting the use of renewable resources and minimizing environmental impact. Future research should conduct life cycle assessments and estimate relevant environmental metrics to fully assess the sustainability and viability of the proposed approaches. Additionally, research should focus on exploring the potential applications of the solubilized lignin and nanoparticles, as well as investigating the scalability and economic viability of these approaches.

## Figures and Tables

**Table 1:** Yield and purity of lignin obtained in the scale-up tests using alkaline (Ak) and deep eutectic solvent (DES) methods. Ref—results from literature reference [3]; different superscript letters represent significant statistical difference (*p* < 0.05).

	Fold	Ak Method	DES Method
Yield (%)	Ref	32 (±6.2) ^a^	50.2 (±2.3) ^a^
	5	31.6 (±4.67) ^a^	43.1 (±3.38) ^b^
	10	32.3 (±1.85) ^a^	35.3 (±1.17) ^c^
	20	34.1 (±4.69) ^a^	32.7 (±0.58) ^c^
Purity (%)	Ref	78.7 (±1.76) ^a^	89.7 (±1.8) ^a^
	5	78.6 (±4.20) ^a^	88.4 (±2.15) ^a^
	10	77.3 (±4.71) ^a^	87.5 (±3.15) ^a^
	20	79.2 (±2.2) ^a^	89.1 (±2.05) ^a^

**Table 2:** Size and PDI of alkaline (AkL) and DES (DESL) lignin nanoparticles prepared using Tween 20 (T20) and Tween 80 (T80), non-concentrated (NC) or concentrated 2- and 5-fold.

		AkL		DESL	
		Size (nm)	PDI	Size (nm)	PDI
T20	NC	304.7 (±2.58)	0.185 (±0.02)	244.7 (±2.94)	0.187 (±0.01)
	2-fold	302.1 (±1.23)	0.152 (±0.02)	257.2 (±3.12)	0.152 (±0.03)
	5-fold	313.2 (±6.60)	0.156 (±0.02)	329.1 (±1.10)	0.308 (±0.03)
T80	NC	298.3 (±0.61)	0.206 (±0.01)	226.9 (±1.23)	0.178 (±0.01)
	2-fold	289.5 (±0.69)	0.180 (±0.01)	234.6 (±0.46)	0.170 (±0.01)
	5-fold	300.6 (±1.97)	0.193 (±0.02)	261.9 (±5.44)	0.209 (±0.01)

**Table 3:** Size (nm), polydispersity index (PDI), and zeta potential (mV) of alkaline (AkL) and DES (DESL) lignin nanoparticles prepared using Tween 20 (T20) and Tween 80 (T80), measured initially and throughout 60 days of storage for the 2-fold concentrated samples. n.d.—not defined.

Sample	Storage Time	Size (nm)	PDI	Zeta Potential (mv)
AkL/T20	Initial	302.1 (±1.23)	0.152 (±0.02)	−26.0 (±0.14)
	15 days	304.8 (±2.88)	0.174 (±0.01)	n.d.
	30 days	308.2 (±6.01)	0.176 (±0.00)	n.d.
	45 days	301.5 (±0.93)	0.167 (±0.01)	n.d.
	60 days	300.8 (±2.05)	0.182 (±0.01)	−25.6 (±0.31)
AkL/T80	Initial	289.5 (±0.69)	0.180 (±0.01)	−30.7 (±1.20)
	15 days	305.0 (±4.33)	0.180 (±0.00)	n.d.
	30 days	301.8 (±4.46)	0.177 (±0.02)	n.d.
	45 days	294.8 (±3.10)	0.191 (±0.01)	n.d.
	60 days	296.3 (±4.40)	0.178 (±0.02)	−16.5 (±0.41)
DESL/T20	Initial	257.2 (±3.12)	0.152 (±0.03)	−27.1 (±0.14)
	15 days	250.2 (±1.02)	0.222 (±0.01)	n.d.
	30 days	252.0 (±10.16)	0.218 (±0.02)	n.d.
	45 days	244.3 (±6.17)	0.196 (±0.03)	n.d.
	60 days	248.7 (±0.91)	0.209 (±0.02)	−22.6 (±0.50)
DESL/T80	Initial	234.6 (±0.46)	0.170 (±0.01)	−33.2 (±0.37)
	15 days	271.1 (±5.99)	0.187 (±0.03)	n.d.
	30 days	234.8 (±3.03)	0.163 (±0.02)	n.d.
	45 days	233.8 (±0.76)	0.171 (±0.01)	n.d.
	60 days	230.3 (±3.51)	0.152 (±0.02)	−27.6 (±0.41)

## Data Availability

No new data were created or analyzed in this study. Data sharing is not applicable to this article.

## Supplementary Materials

The following supporting information can be downloaded at: https://www.mdpi.com/article/10.3390/foods14244274/s1, Figure S1: Calibration curves of lignin dissolution (alkaline and DES) using DMSO as solvent.
