# Supplementary figures and images for: From Grape Stalks to Lignin Nanoparticles: A Study on Extraction Scale-Up, Solubility Enhancement and Green Nanoparticle Production
Source: Foods. 2025 Dec 12;14(24):4274. doi: 10.3390/foods14244274 (PMC12731988; doi:10.3390/foods14244274)

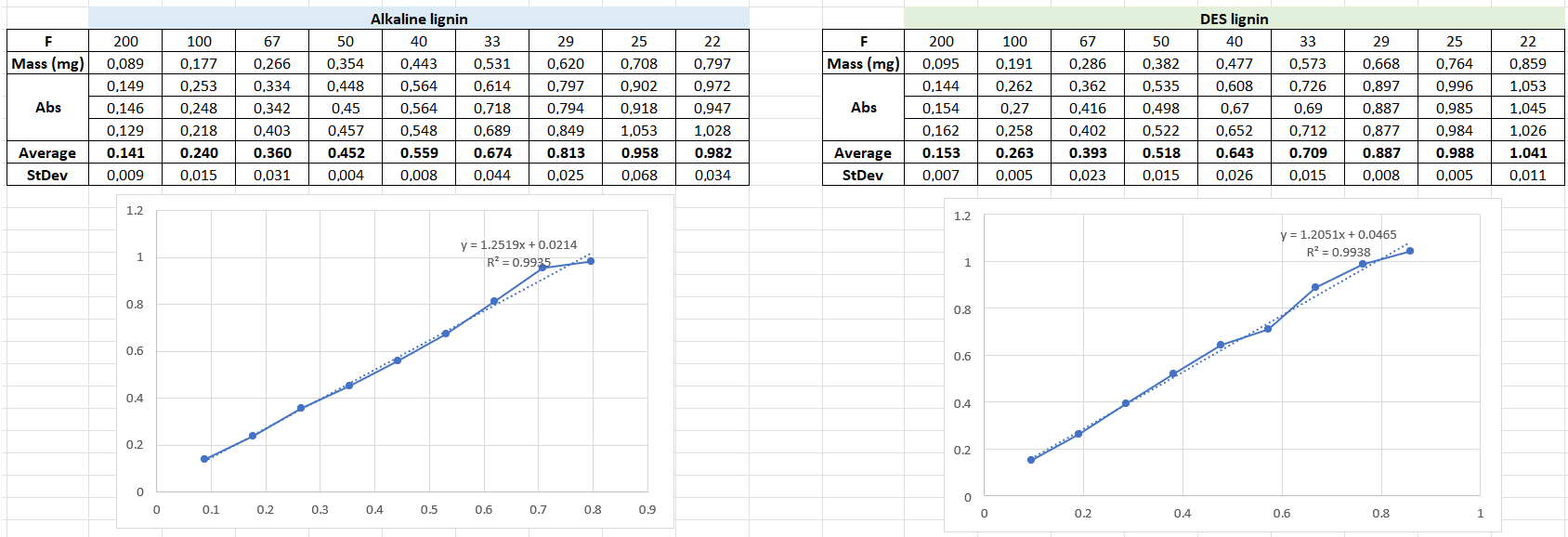

Supplement: Supplementary file 1 [file foods-14-04274-s001.zip › foods-3921562-supplementary.png]
